# The m^6^A methylation and expression profiles of mouse neural stem cells after hypoxia/reoxygenation

**DOI:** 10.1186/s13287-024-03658-8

**Published:** 2024-02-16

**Authors:** Shaoqiong Zhang, Kaile Cui, Yuanyuan Li, Yiting Fan, Dongxu Wang, Xingen Yao, Bo Fang

**Affiliations:** Department of Anesthesiology, The First Hospital of China Medical University, Shenyang, China

**Keywords:** Neural stem cell, Hypoxia/reoxygenation, N6-methyladenosine methylation, Proliferation, Migration, Differentiation

## Abstract

**Background:**

Ischemia–reperfusion injury to the central nervous system often causes severe complications. The activation of endogenous neural stem cells (NSCs) is considered a promising therapeutic strategy for nerve repair. However, the specific biological processes and molecular mechanisms of NSC activation remain unclear, and the role of N6-methyladenosine (m^6^A) methylation modification in this process has not been explored.

**Methods:**

NSCs were subjected to hypoxia/reoxygenation (H/R) to simulate ischemia–reperfusion in vivo. m^6^A RNA methylation quantitative kit was used to measure the total RNA m^6^A methylation level. Quantitative real-time PCR was used to detect methyltransferase and demethylase mRNA expression levels. Methylated RNA immunoprecipitation sequencing (MeRIP-seq) and RNA sequencing (RNA-seq) were conducted for NSCs in control and H/R groups, and the sequencing results were analyzed using bioinformatics. Finally, the migration ability of NSCs was identified by wound healing assays, and the proliferative capacity of NSCs was assessed using the cell counting kit-8, EdU assays and cell spheroidization assays.

**Results:**

Overall of m^6^A modification level and *Mettl14* mRNA expression increased in NSCs after H/R treatment. The m^6^A methylation and expression profiles of mRNAs in NSCs after H/R are described for the first time. Through the joint analysis of MeRIP-seq and RNA-seq results, we verified the proliferation of NSCs after H/R, which was regulated by m^6^A methylation modification. Seven hub genes were identified to play key roles in the regulatory process. Knockdown of *Mettl14* significantly inhibited the proliferation of NSCs. In addition, separate analysis of the MeRIP-seq results suggested that m^6^A methylation regulates cell migration and differentiation in ways other than affecting mRNA expression. Subsequent experiments confirmed the migration ability of NSCs was suppressed by knockdown of *Mettl14*.

**Conclusion:**

The biological behaviors of NSCs after H/R are closely related to m^6^A methylation of mRNAs, and *Mettl14* was confirmed to be involved in cell proliferation and migration.

**Supplementary Information:**

The online version contains supplementary material available at 10.1186/s13287-024-03658-8.

## Background

Ischemia–reperfusion injury is a common pathophysiological process in many diseases including ischemic stroke, myocardial infarction, acute renal injury, and hemorrhagic shock [1]. In addition, some surgical techniques require that blood supply to organs must be blocked during the perioperative period, resulting in ischemia–reperfusion injury such as cerebral ischemia–reperfusion injury after carotid endarterectomy and spinal cord ischemia–reperfusion injury after aortic surgery [2, 3]. Local neurons are lost, myelin sheaths are severely degraded, the barrier is damaged, and edema occurs after central nervous system ischemia–reperfusion injury [4–7]. Ischemia–reperfusion causes dramatic changes in the internal environment on which cells depend for survival. Hypoxia/reoxygenation (H/R) is one of the most common and detrimental internal environmental changes experienced by local cells and is often used to simulate the process of ischemia–reperfusion in vitro [8–10].

Neural stem cells (NSCs) are multipotent stem cells that can self-renew and differentiate into different cell types in the central nervous system [11]. At present, there are two strategies to treat degenerative nervous system diseases: NSC transplantation and endogenous NSC activation [11]. However, complications such as immune rejection and tumorigenicity of the former hinder its popularization [12, 13]. Adult NSCs, which are originally in a static state, begin to undergo a transformative biological process known as endogenous NSC activation under the state of injury or stress [11]. There is no doubt that the proliferation of NSCs is the basis of nerve tissue repair, and many studies have also reported that endogenous NSC proliferation occurs after ischemic stroke [14–17]. However, neural repair is a complex process that goes beyond normal proliferation. NSCs must be endowed with more capabilities, such as the ability to migrate to the injured site and successfully differentiate into the correct neuronal subtype. Therefore, we used bioinformatics analysis to explore the biological processes of NSCs after H/R as comprehensively as possible.

N6-methyladenosine (m^6^A) refers to the methylation of the adenosine base at the nitrogen-6 position, which is the most common and abundant RNA molecular modification in eukaryotes. The presence of methyltransferases and demethylases makes the modification dynamically reversible. Regulation of m^6^A methylation of mRNA regulates gene expression and therefore the biological processes of NSCs. Loss of fat mass and obesity-associated gene (*FTO*), a demethylase gene, reduced proliferation and neuronal differentiation of adult NSCs in vivo [18]. After knockout of methyltransferase-like 14 (*Mettl14*), the proliferation of embryonic NSCs was significantly inhibited and early differentiation occurred [19]. In this study, we aimed to analyze the m^6^A methylation profiles of NSCs after H/R for the first time and to reveal the molecular mechanism by which m^6^A methylation regulates the activation of NSCs. In addition, because the molecular mechanism of endogenous activation of NSCs remains unclear, we explored the hub genes that regulate the biological activities of NSCs after H/R by combining mRNA m^6^A methylation and expression analyses.

## Materials and methods

### Cell culture and H/R

C17.2 NSCs were purchased from Meisen Chinese Tissue Culture Collections (Jinhua, Zhejiang, China) and cultured in Dulbecco’s modified Eagle’s medium with 10% fetal bovine serum, 1% penicillin–streptomycin, and 1% glutamine (Procell Life Science & Technology Co., Ltd, Wuhan, Hubei, China). The culture medium was replaced every two days, and the cells were passaged every 4–5 days. To simulate ischemia–reperfusion in vitro, NSCs were placed in a humid anaerobic chamber (MC-101 model, Billups-Rothenberg Inc., Del Mar, CA, USA) perfused with 5% O_2_, 5% CO_2_, and 90% N_2_ at 37 °C for 12 h. NSCs were then moved to standard oxygen conditions and cultured for 48 h. The cells in the control group were cultured under standard oxygen conditions.

### m^6^A RNA methylation quantification

Total RNA was extracted using TRIzol (Takara Bio Inc., Shiga, Japan). The total RNA m^6^A methylation level was detected via EpiQuik m^6^A RNA Methylation Quantification Kit (P-9005; Epigentek Group Inc., Farmingdale, NY, USA). The brief operation process is as follows: 2 μL control (negative or positive) and 200 ng sample RNA were added into the eight tubes in the kit, respectively, and the total RNA was bound to the orifice plate with high-efficiency RNA binding solution. After adding the develop solution and stop solution, the absorption value at 450 nm was read with the microplate reader.

### Quantitative real-time PCR (qRT-PCR)

Total RNA was isolated after the cells were lysed using TRIzol reagent (Takara Bio Inc., Otsu, Shiga, Japan). The reverse transcribed RNA was equal to 450 ng using the cDNA Reverse Transcription Kit (Takara Bio Inc., Otsu, Shiga, Japan), according to the manufacturer’s instructions. Target genes were amplified using the SYBR Green method with a real-time fluorescent quantitative PCR kit (Takara Bio Inc., Otsu, Shiga, Japan). mRNA levels were quantified relative to those of glyceraldehyde phosphate dehydrogenase (GAPDH) as a reference gene. The primer sequences used are listed in Table 1.

**Table 1:** Primer sequence in quantitative reverse-transcription polymerase chain reaction

Gene	Sequence
*Mettl14*^a^	F: 5′-TCACCTCCTCCCAAGTCCAAGTC-3′
	R: 5′-CCCTAAAGCCACCTCTCTCTCCTC-3′
*Mettl3*^b^	F: 5′-CGCTGCCTCCGATGTTGATCTG-3′
	R: 5′-TCTCCTGACTGACCTTCTTGCTCTG-3′
*WTAP*^c^	F: 5′-ACGCAGGGAGAACATTCTTGTCATG-3′
	R:5′-TCGGCTGCTGAACTTGCTTGAG-3′
*FTO*^d^	F: 5′-CTCACAGCCTCGGTTTAGTTCCAC-3′
	R: 5′-CGTCGCCATCGTCTGAGTCATTG-3
*ALKBH5*^e^	F: 5′-CGGGACCACCAAGCGGAAATAC-3′
	R: 5′-CCTCTTCCTCCTTCTGCAACTGATG-3′
*GAPDH*^f^	F: 5′-GGCAAATTCAACGGCACAGTCAAG-3′
	R: 5′-TCGCTCCTGGAAGATGGTGATGG-3′

^a^methyltransferase like 14, ^b^methyltransferase like 3, ^c^WT1 associating protein, ^d^fat mass and obesity-associated gene, ^e^alkB homolog 5, ^f^glyceraldehyde phosphate dehydrogenase

### Methylated RNA immunoprecipitation sequencing (MeRIP-seq) and data analysis

The m^6^A-IP-Seq service was outsourced to CloudSeq Biotech Inc. (Shanghai, China). Immunoprecipitation of total RNA was completed using the GenSeq^®^ m^6^A-IP Kit (GenSeq Inc., Shanghai, China) according to the manufacturer’s instructions. Briefly, RNA was randomly fragmented to approximately 200 nucleotides using RNA fragmentation reagents. Protein A/G beads were rotated at room temperature (25 °C) for 1 h with m^6^A antibodies to achieve coupling. The A/G bead-coupled antibodies were then incubated with the RNA fragments and further rotated at 4 °C for 4 h. Following incubation, the resulting complexes were washed multiple times. The captured RNA was eluted from the complexes and purified. A GenSeq^®^ Low Input Whole RNA Library Prep Kit (GenSeq, Inc., Shanghai, China) was used to construct RNA libraries for IP and input samples according to the manufacturer’s instructions. An Agilent 2100 Bioanalyzer (Agilent Technologies Inc., Palo Alto, CA, USA) was used to identify libraries which were then sequenced on a NovaSeq platform (Illumina, San Diego, CA, USA). Paired-end reads were collected from an Illumina NovaSeq 6000 sequencer (Illumina, San Diego, CA, USA), and quality was controlled by Q30. Then, 3′ adaptor trimming and low-quality reads were removed using cutadapt software (v1.9.3) [20]; STAR software and Hisat2 software (v2.0.4) were used to match the clean reads of the input libraries, and all libraries, respectively, to the reference genome (UCSC MM10) [21, 22]. Methylated sites on RNAs (peaks) were identified using MACS software [23]. Differentially methylated sites were identified using diffReps [24]. These peaks on the exons of mRNAs were screened using our own program, and the corresponding annotations were completed using this program. The foldchange cutoff was 2, and the *P* value cutoff was 0.00001.

### RNA sequencing (RNA-seq) and data analysis

The GenSeq^®^ rRNA Removal Kit (GenSeq, Inc., Shanghai, China) was used to remove rRNAs from the samples according to the manufacturer’s instructions. After rRNAs were removed from the samples, a sequencing library was constructed using the GenSeq^®^ Low Input RNA Library Prep Kit (GenSeq, Inc., Shanghai, China) according to the manufacturer’s instructions. The BioAnalyzer 2100 system (Agilent Technologies, Inc., Palo Alto, CA, USA) was used to control the quality of and quantify the libraries, and an Illumina NovaSeq 6000 sequencer (Illumina, San Diego, CA, USA) was used for library sequencing with 150 bp paired-end reads. Next, the paired-end reads were harvested and quality-controlled by Q30. We used cutadapt software (v1.9.3) to remove 3′ adaptor-trimming and low-quality reads [20], and hisat2 software (v2.0.4) to match high-quality reads to the reference genome [22]. HTSeq software (v0.9.1) was used to obtain the raw count at the gene level as the expression profiling; edgeR (v3.16.5) was used to perform normalization, and differentially expressed mRNAs were identified by *P* value and foldchange [25]. The foldchange cutoff was 2, and the *P* value cutoff was 0.05.

### Bioinformatics analysis

An idiographic online tool (http://rtools.cbrc.jp/idiographica/) was used to present differential methylation sites on RNAs transcribed from genes on each chromosome [26]. Gene Ontology (GO) enrichment analysis and Kyoto Encyclopedia of Genes and Genomes (KEGG) pathway analysis of mRNAs with m^6^A methylation differences or expression differences were performed using R software 4.2.1 (R Foundation for Statistical Computing, Vienna, Austria). We determined the interaction relationship between biomolecules using the STRING database (https://string-db.org/). Then, Cytoscape software 3.9.1 (The Cytoscape Consortium, San Diego, CA, USA) was used to construct the protein–protein interaction (PPI) network and select the hub genes.

### Cell transfection

To inhibit the expression of *Mettl14*, C17.2 NSCs were uniformly cultured in a six-well plate at a density of 5 × 10^5^ cells per well. After the cells were attached to the wall, si-Mettl14 (20 µM) and the negative control (NC) (20 µM) (RiboBio, Guangzhou, Guangdong, China) were incubated with transfection reagents and then added to the medium. After 48 h, a transfection efficiency of 80% was observed in the fluorescent control group.

### Wound healing assay

The cells were laid out in a labeled six-well plate at a density of 4 × 10^5^ per well and cultured overnight, using 200 μL pipette to vertically scratch the single-layer cells. The scratched cells were removed by PBS washing for three times. The cell images were taken under the optical microscope 48 h after H/R.

### Western blot

The adherent cells were harvested in RIPA lysis buffer (Beyotime Biotechnology, Shanghai, China) on ice. The lysed cells were scraped and transferred to 1.5 mL Eppendorf tubes. After 10 min incubated on ice, centrifuge at 4 °C at 12,000 rpm for 15 min. Gently absorb the supernate and transfer it to a newly pre-cooled centrifuge tube. Total protein concentration was determined by bicinchoninic acid (BCA) protein assay kit (Beyotime Biotechnology, Shanghai, China). The protein loading buffer (Beyotime Biotechnology, Shanghai, China) was then added and heated in a boiling water bath at 100 °C for 5 min to denature the protein. A protein sample of 30 μg was isolated by SDS-PAGE adhesive, and membrane transfer was conducted through polyvinylidene fluoride (PVDF, Millipore, Boston, MA, USA) membrane. The membrane was sealed with 5% skim milk at normal temperature for 2 h and then incubated with specific primary antibody at 4 °C overnight. The affinity purified Goat Anti-Rabbit Mouse IgG antibody (Abmart, Shanghai, China) was then used to incubate the membrane at room temperature for 1 h The protein bands were mixed with ECL solvent, and images were collected with the enhanced chemiluminescence (Thermo Fisher Scientific, Waltham, MA, USA).

### Cell counting kit-8 (CCK-8) assay

si-Mettl14- and NC-transfected NSCs in the logarithmic growth phase were seeded in 96-well plates. Cells were counted 24 h in advance to ensure a density of 5 × 10^3^ per well. Next, 10% CCK-8 (Apexbio, Houston, TX, USA) was added and incubated at 37 °C for 2 h. The optical density (OD) of the solution in each sample was measured spectrophotometrically at 450 nm. The experiment was repeated three times for statistical analysis.

### EdU assay

The EdU assay for NSC proliferation was performed after 24 h of reoxygenation using a commercial kit (Beyotime Biotech, Shanghai, China). Cells were cultured in a medium containing 10 μM EdU for 24 h according to the manufacturer’s instructions. Then, 4% paraformaldehyde was used for cell fixation and 0.3% Triton X-100 (Sigma-Aldrich, St Louis, MO, USA) was used to permeabilize the cells. Cells were stained with the included click additive solution, and DAPI (Servicebio, Wuhan, Hubei, China) was used to perform nuclear staining. Finally, images were captured using fluorescence microscopy. The percentage of EdU-labeled cells was counted using ImageJ software 1.48 (National Institutes of Health, Bethesda, MD, USA).

### Isolation, culture of primary NSCs

C57BL/6 mice weighing over 30 g, aged 11 weeks, with a first pregnancy and 16 days of gestation (Vital River, Beijing, China) were purchased for the isolation of primary NSCs. Briefly, the brain tissue was isolated from fetal mice in pregnant mice and cut into about 1 mm^3^ tissue blocks with iris scissors, which digested by 0.125% trypsin, treated at 37 °C for 20 min, and shaken 2–3 times during the period. Discarding the supernatant, the complete medium was added to terminate digestion. After NSCs was washed with PBS and centrifuged, they were cultured in T25 cell culture bottle with complete medium at 37 °C and 5% CO_2_ incubator for 4-6 h, and replaced with serum-free culture medium. The medium was changed every 2 days, and the cells were cultured for 3–4 days for passaging. The above animal experimental steps were conducted in accordance with the Guide for the Care and Use of Laboratory Animals published by the US National Institutes of Health. All animal experimental procedures were reviewed and approved by the Ethics Committee of the Animal Department of China Medical University (Approval number: CMUXN2023046).

### Cell spheroidization assays

Primary cells were cultured in serum-free medium. The cells of logarithmic growth stage were collected, inoculated at a density of 1000 cells/well to a low-adhesion 6-well plate. After stationary culture 7–10 days, the suspended neurosphere with a count of more than 50 cells was observed under the microscope and its diameter was assessed.

### Statistical analysis

The results of the experimental data were expressed as mean ± standard error of the mean (SEM) and were analyzed and plotted using GraphPad Prism 9 (GraphPad Software Inc., La Jolla, CA, USA). The Shapiro–Wilk test was used to determine whether the data were normally distributed (*P* > 0.05, normal distribution). When the two groups of data were normally distributed and had homogeneous variances, an unpaired t test was used for statistical analysis. Welch’s t test was used when the variance was inconsistent. In addition, if the data did not follow a normal distribution, a Mann–Whitney test was used to evaluate the differences. Differences were considered significant when *P* < 0.05.

## Results

### Overall of m^6^A modification level and ***Mettl14*** mRNA expression increased in NSCs after H/R treatment

**Fig. 1:**
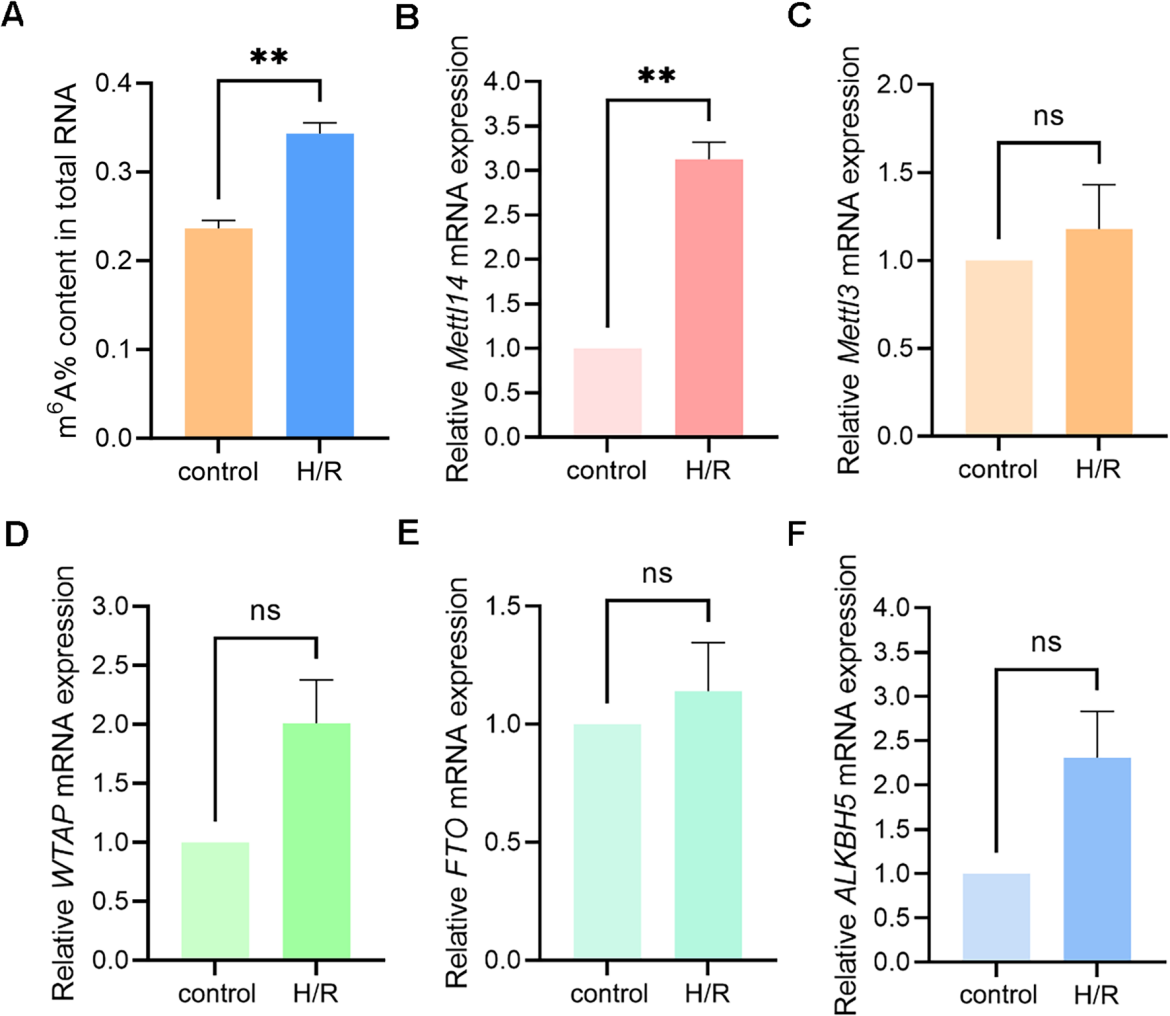
Overall m^6^A modification level and mRNA expression of five methyltransferases or demethylases in NSCs after H/R. **A** Overall m^6^A modification level in NSCs increased after H/R. **B** qRT-PCR detected that *Mettl14* mRNA expression in NSCs of H/R group was significantly higher than that of control group. **C**–**F** The results of qRT-PCR showed that there was no significant difference in the mRNA expression of METTL3, WTAP, FTO, and ALKBH5 between the control group and the H/R group (n = 3). **P < 0.01; ns indicates *P* > 0.05. The data are presented as the mean ± SEM

After H/R treatment, the overall m^6^A methylation modification changes of mRNAs in NSCs were detected. It was confirmed that more m^6^A methylated modification of mRNAs occurred in NSCs after H/R (*P* = 0.0020) (Fig. 1A). To investigate the causes of mRNA m^6^A methylation changes in NSCs after H/R, we evaluated the RNA expression levels of three methyltransferases, METTL14, methyltransferase-like 3 (METTL3), WT1 associating protein (WTAP), and two demethylases FTO, and alkB homolog 5 (ALKBH5), using qRT-PCR. However, after H/R, only increased mRNA expression of METTL14 was observed in NSCs (*P* = 0.0081) (Fig. 1B), and there was no significant difference in mRNA expression of METTL3, WTAP, FTO, and ALKBH5 (*P* = 0.5519,* P* = 0.1105,* P* = 0.5640,* P* = 0.1285, respectively) (Fig. 1C–F).

### Overview of m^6^A methylated RNA immunoprecipitation sequencing results and topological distribution of m^6^A methylation peaks

**Fig. 2:**
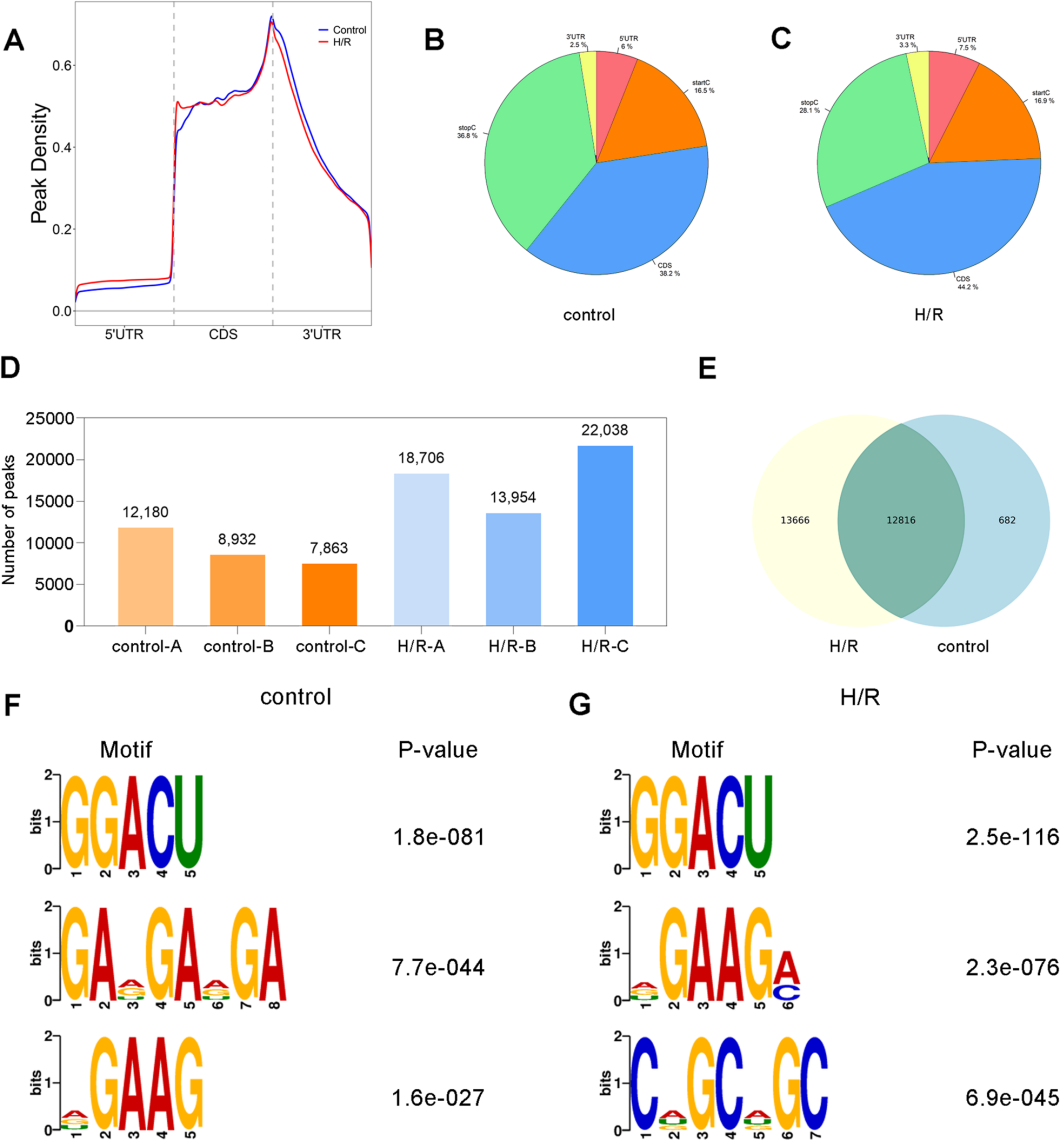
Topological distribution of m^6^A methylation peaks before and after H/R treatment. **A** The distribution of m^6^A methylation peaks on RNAs of NSCs in control and H/R groups in gene structures. **B**, **C** Distribution percentage of m^6^A methylation peaks on RNAs in each gene structure in control group and H/R group. **D** The number of m^6^A methylation peaks of each sample in control group and H/R group. **E** Coincidence and difference of m^6^A methylation peaks in control group and H/R group. **F**, **G** The three most conservative m^6^A methylation motifs in control group and H/R group, respectively

To compare the differences in mRNA m^6^A methylation in NSCs after H/R treatment, we implemented MeRIP-Seq. Average raw reads (Additional file 1: Table S1) of 89.84 Mb and 91.27 Mb were obtained from IP samples and input samples, respectively. After 3′ adapter trimming and low-quality read removal, clear reads of IP samples and input samples were obtained. An average of 44.99% of the reads were mapped to the reference genome (Additional file 2: Table S2). We eliminated multiple mapped reads (1.74%), and the remaining unique mapped reads were used for subsequent analyses (43.25%) (Additional file 2: Table S2). After comparing the sequencing data of IP and input samples, we annotated the m^6^A peak distribution. The m^6^A methylation peaks in the control and H/R groups were mainly distributed in the CDS region. In particular, after H/R treatment, the proportion of m^6^A peaks in the CDS region further increased from 38.2 to 44.2% (Fig. 2A–C). We identified m^6^A peaks in mRNAs in each sample of the control and H/R groups (Additional file 3: Table S3). Specifically, 9658 peaks were identified in the control group on average, while average of 18,233 peaks were identified in the H/R group (Fig. 2D). There were 12,816 overlapping peaks between the two groups, accounting for 47.2% of the total peaks (Fig. 2E). In addition, Fig. 2F, G shows the three most conserved motifs in the control and H/R groups, respectively.

### Significant changes in m^6^A methylation after H/R treatment

**Fig. 3:**
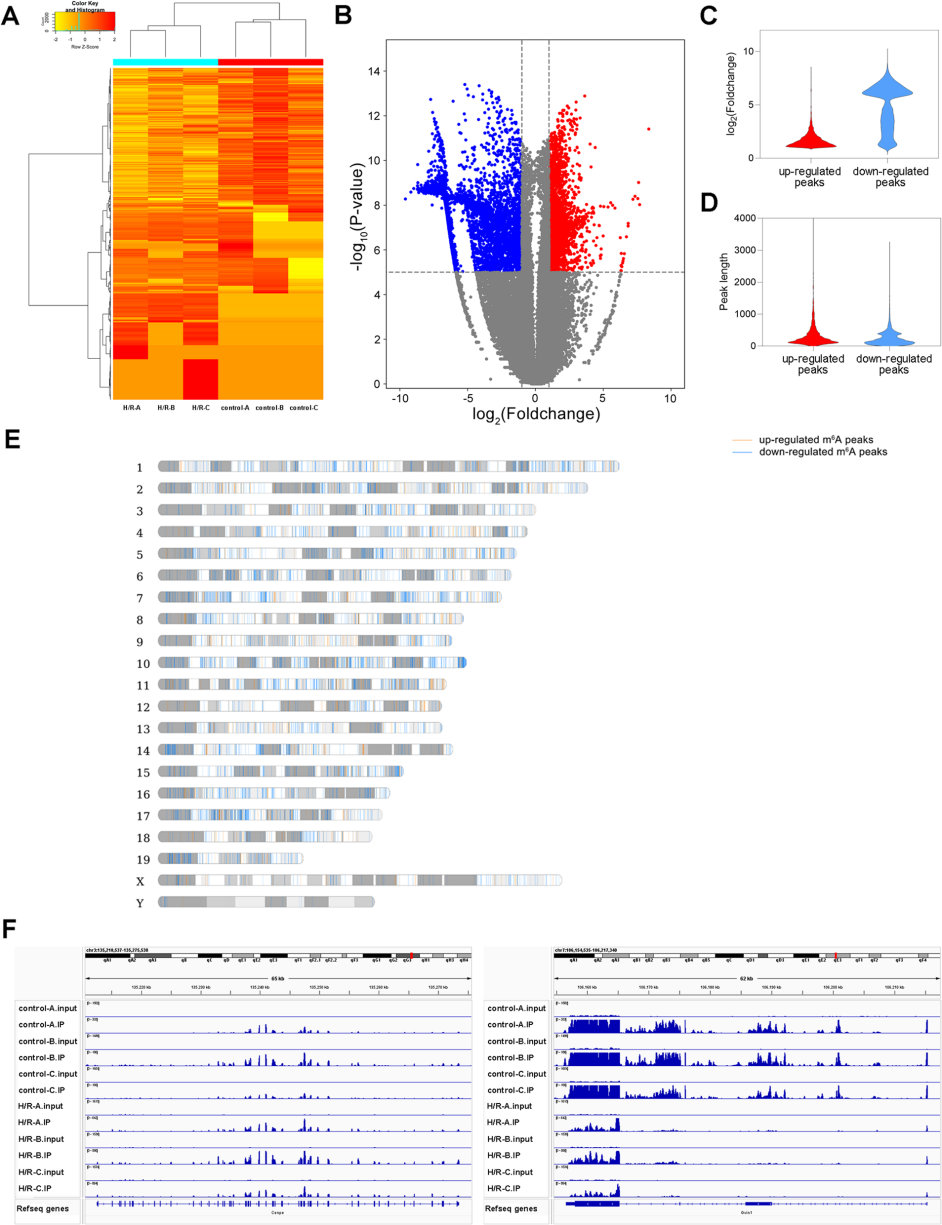
Difference of m^6^A methylation peaks between control group and H/R group. **A** Heat map showing m^6^A methylation difference of samples in control group and H/R group. **B** Volcanic plot showing significantly up-regulated (red) and down-regulated (blue) m^6^A methylation peaks (Foldchange cutoff was 2, *P* value cutoff was 0.00001). **C** Foldchanges of up-regulated and down-regulated m^6^A methylation. **D** Length of up-regulated and down-regulated m^6^A methylation peaks. **E** Chromosome distribution map of differential m^6^A methylation sites. **F** IGV plots showing *Cenpe* and *Gvin1* m^6^A methylation sites, respectively

Compared with the control group, 8540 peaks in the H/R group demonstrated changes, including 2847 upregulated peaks and 5693 downregulated peaks (Additional file 4: Table S4, Fig. 3A, B). The average logarithmic foldchange of upregulated peaks was 1.70, and that of downregulated peaks was 4.85 (Fig. 3C). The average lengths of the upregulated and downregulated peaks were 341 and 245 bp, respectively (Fig. 3D). Chr10 had the largest number of differential peaks of mRNAs transcribed from the gene, and the number of differential methylation sites was 673 (Fig. 3E). In addition, these differentially expressed peaks came from 6200 mRNAs (Additional file 4: Table S4). Details of the 20 methylation peaks with the most significant differences are presented in Table 2. Figure 3F shows the two mRNAs with the largest number of m^6^A methylation peaks, *Cenpe* and *Gvin1*.

**Table 2:** The top 20 significantly changed m^6^A methylation peaks based on *P* value

Peak ID	Peak start	Peak end	Foldchange	*P* value	Regulation	Chromosome	Gene name
diffreps_peak_677001	109,575,127	109,575,520	12.42056075	1.29283E−13	Up	chr8	*Hp*
diffreps_peak_468654	135,259,978	135,260,160	8.022988506	2.44216E−13	Up	chr3	*Cenpe*
diffreps_peak_280810	11,447,307	11,447,500	5.090078329	3.64478E−13	Up	chr16	*Snx29*
diffreps_peak_643379	143,740,269	143,740,360	5.496202532	4.4459E−13	Up	chr7	*Osbpl5*
diffreps_peak_117541	40,714,710	40,714,720	7.885802469	5.55243E−13	Up	chr11	*Hmmr*
diffreps_peak_557960	138,187,041	138,187,226	4.2890625	5.66926E−13	Up	chr5	*Taf6*
diffreps_peak_34656	136,486,271	136,486,444	4.059471366	5.69005E−13	Up	chr1	*Kif14*
diffreps_peak_120760	50,292,241	50,292,336	4.255172414	5.82923E−13	Up	chr11	*Maml1*
diffreps_peak_285637	22,985,851	22,986,068	5.007760532	7.25728E−13	Up	chr16	*Kng2*
diffreps_peak_614902	35,409,580	35,409,780	3.795546559	7.67188E−13	Up	chr7	*Cep89*
diffreps_peak_44561	173,736,186	173,736,471	36.87654321	3.98127E−14	Down	chr1	*AI607873*
diffreps_peak_425981	160,891,995	160,892,388	17.7902439	4.5173E−14	Down	chr2	*Lpin3*
diffreps_peak_675357	105,292,334	105,292,445	32.83950617	6.29123E−14	Down	chr8	*Exoc3l*
diffreps_peak_557702	137,730,882	137,731,180	10.6686217	7.08389E−14	Down	chr5	*Nyap1*
diffreps_peak_18578	74,881,508	74,882,040	9.29040404	8.4985E−14	Down	chr1	*Fev*
diffreps_peak_175511	116,143,668	116,143,741	9.484777518	9.83761E−14	Down	chr12	*Vipr2*
diffreps_peak_443517	51,388,701	51,389,080	14.7392638	1.0669E−13	Down	chr3	*Mgarp*
diffreps_peak_549149	114,904,981	114,905,076	30.97777778	1.19502E−13	Down	chr5	*Oasl2*
diffreps_peak_210970	119,494,901	119,495,220	9.419786096	1.34714E−13	Down	chr13	*Gm7120*
diffreps_peak_620447	55,930,901	55,931,100	23.88571429	1.40611E−13	Down	chr7	*Cyfip1*

### Enrichment analysis of genes with m^6^A methylation differences after H/R treatment

**Fig. 4:**
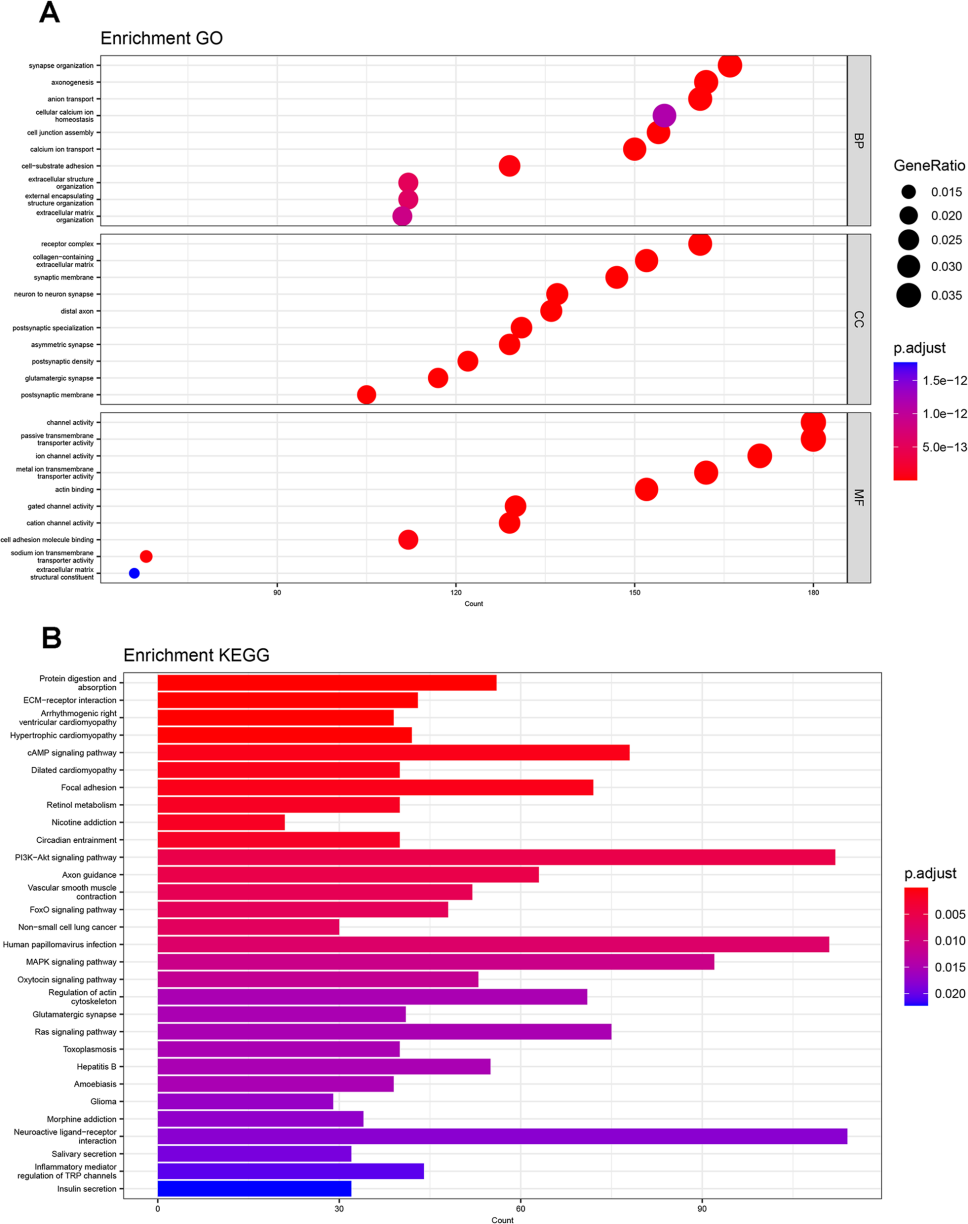
Enrichment analysis of the source genes of mRNAs with m^6^A methylation changes in NSCs after H/R. **A** 30 terms with the largest number of enriched genes from biological processes, cell components and molecular functions by GO enrichment analysis. **B** 30 pathways with the largest number of enriched genes by KEGG pathway analysis

**Fig. 5:**
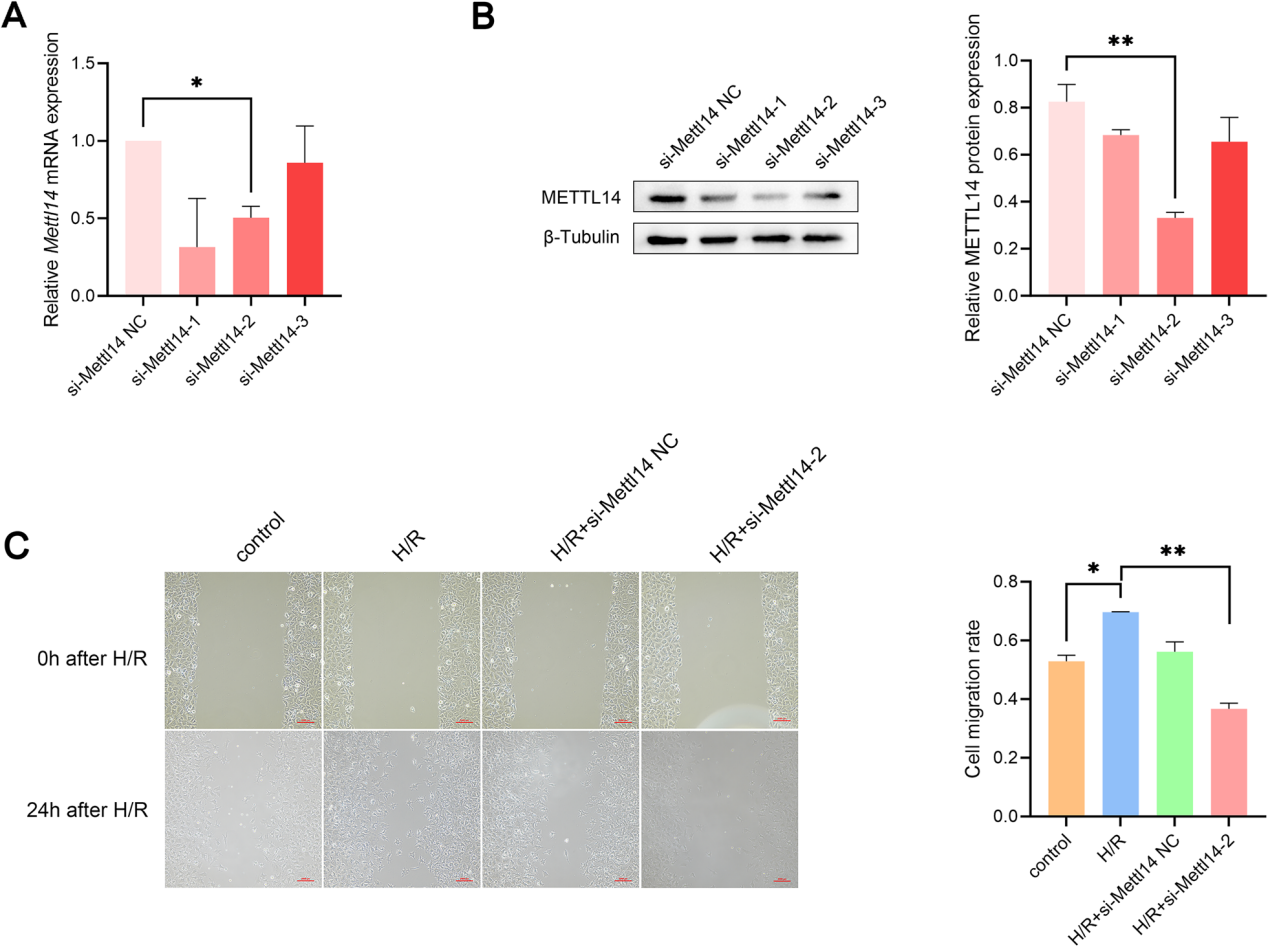
Knockdown of *Mettl14* inhibited the migration of NSCs after H/R. **A** Detection of knockdown efficiency of si-Mettl14 by qRT-PCR (n = 3). *P < 0.05. **B** Detection of knockdown efficiency of si-Mettl14 by western blotting (n = 3). **P < 0.01. Full-length blots are presented in Additional file 15: Fig. S2. **C** Detection of the migration capability of NSCs that undergo H/R treatment after *Mettl14* knockdown by wound healing assays (n = 3). *P < 0.05; **P < 0.01

After duplication of mRNAs according to gene symbols, 5533 mRNAs with m^6^A methylation differences were obtained. GO analysis showed that 2688 terms were enriched (Additional file 5: Table S5) including biological processes, cell components, or molecular functions. The 10 terms with the largest number of enriched genes in all aspects are shown in Fig. 4A. The analysis results showed that mRNAs with m^6^A methylation differences after H/R were most closely associated with biological processes such as synapse organization, cellular ion homeostasis, and extracellular structure organization. In terms of molecular functions, they were associated with ion transport and cell adhesion. The genes enriched in synapse organization, extracellular structure organization, and cell adhesion molecule binding terms in GO analysis results are shown in Additional file 6: Fig. S1A–C. In KEGG analysis, mRNAs with differential methylation of m^6^A following H/R were enriched in 42 pathways (Additional file 7: Table S6). Similar to the GO analysis results, it could be concluded that these mRNAs participate in synaptic and extracellular matrix functions and were mainly enriched in classical signaling pathways, such as cAMP, PI3K-Akt, FOXO, and MAPK (Fig. 4B). Additional file 6: Fig. S1D, E shows the genes enriched in the ECM-receptor interaction and axon guidance terms in the KEGG analysis results. Guided by the above results, we validated the migration ability of NSCs after H/R treatment and explored the role of *Mettl14* in the migration of NSCs. Knockdown efficiency of si-Mettl14 was validated using qRT-PCR and western blotting techniques (Fig. 5A, B). The migration rate of NSCs in the H/R group was higher than that in the control group (*P* = 0.0141, Foldchange = 1.3164), and the enhanced migration ability was eliminated after knocking down *Mettl14* (*P* = 0.0033) (Fig. 5C).

### Gene expression changes and enrichment analysis after H/R treatment

**Fig. 6:**
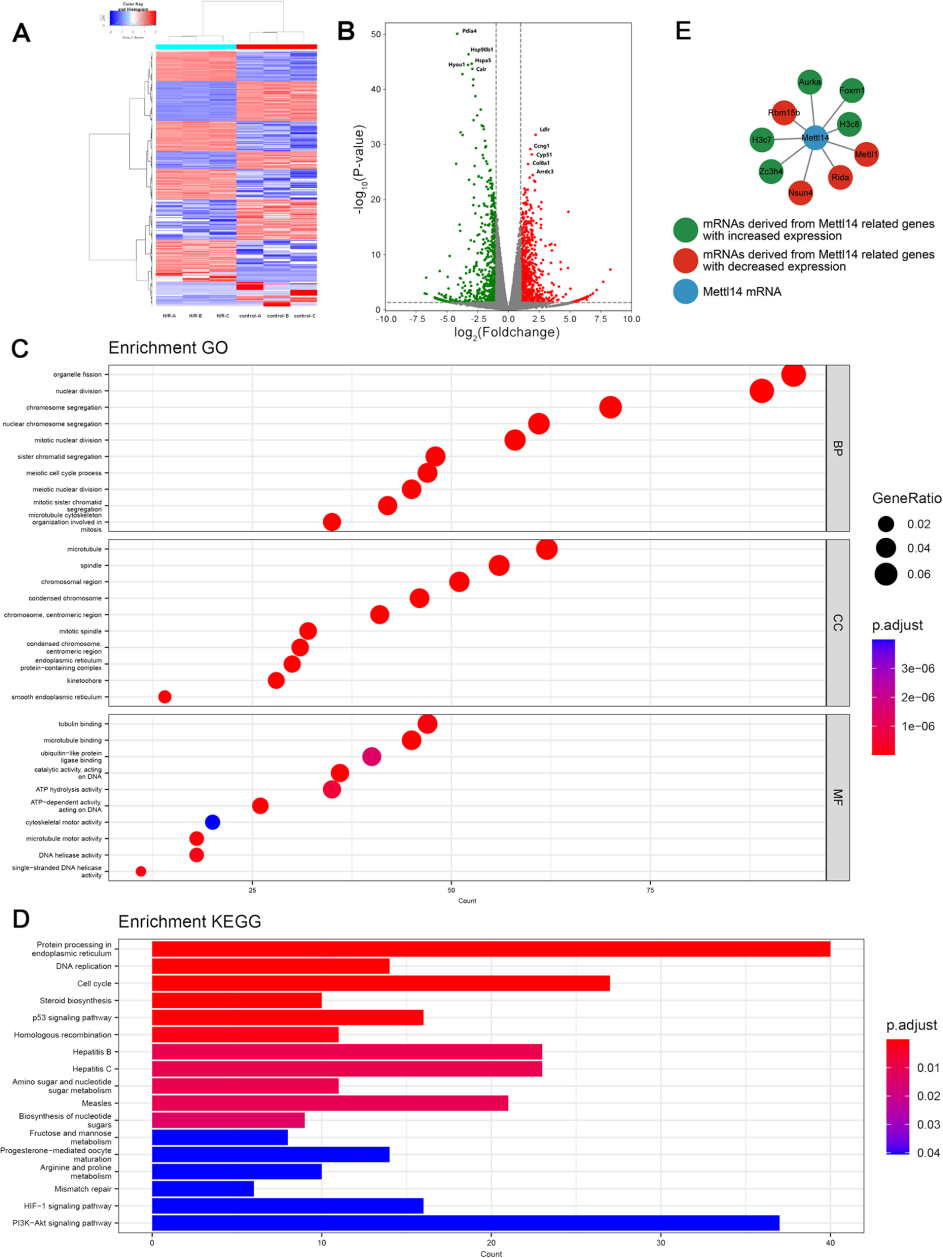
Difference of mRNA expression between control group and H/R group. **A** Heat map showing the difference of mRNA expression of samples in control group and H/R group. **B** Volcanic plot showing mRNAs with significantly up-regulated (red) and down-regulated (green) expression (Foldchange cutoff was 2, *P* value cutoff was 0.05). **C**, **D** GO and KEGG analysis of the source genes of mRNAs with differential expression. **E** Differentially expressed genes associated with methyltransferase METTL14 from the STRING database

To further explore the effect of m^6^A methylation differences on gene expression after H/R treatment, we conducted RNA sequencing. A total of 1454 differentially expressed mRNAs were identified, including 755 genes with increased expression and 699 genes with decreased expression (Additional file 8: Table S7; Fig. 6A, B). The gene symbols of the 20 mRNAs with the most significant differences in expression are shown in Table 3. 1454 differentially expressed mRNAs were enriched in 960 GO terms (Additional file 9: Table S8). The results showed that these mRNAs were closely related to the biological process of cell division, and from the perspective of cell component analysis these mRNAs also played a role in the chromosome structure (Fig. 6C). For their own molecular functions, they not only play a role related to DNA helicase, but also participate in ATP hydrolysis and cytoskeleton movement (Fig. 6C). KEGG analysis showed that these mRNAs were enriched in 17 pathways (Additional file 10: Table S9), all of which are shown in Fig. 6D. Among them, DNA replication and the cell cycle were more prominent. The p53, HIF-1, and PI3K-Akt signaling pathways have also been identified. In addition, we attempted to use the STRING database to identify the parts associated with the key methyltransferase METTL14 among these 1454 differentially expressed mRNAs, as shown in Fig. 6E.

**Table 3:** The top 20 mRNAs with the most significant difference in expression based on *P* value

mRNA	*P* value	Log_2_(Foldchange)	Regulation
*Ldlr*	1.83434E−32	2.207904365	Up
*Ccng1*	7.23984E−30	1.786140865	Up
*Cyp51*	6.80734E−29	1.896848422	Up
*Col8a1*	3.76642E−27	1.597776827	Up
*Arrdc3*	3.68915E−25	1.978763327	Up
*Dhcr24*	1.09436E−24	1.733040131	Up
*Glt8d2*	3.94714E−24	2.095644657	Up
*Acat2*	6.01479E−24	2.176427662	Up
*Fstl1*	1.03499E−22	1.301807839	Up
*Adamts4*	1.62531E−22	1.578441346	Up
*Pdia4*	8.62605E−51	− 4.15748117	Down
*Hsp90b1*	4.71166E−47	− 3.235316509	Down
*Hspa5*	2.42181E−45	− 2.987004814	Down
*Hyou1*	3.96329E−45	− 3.277397079	Down
*Calr*	1.98337E−44	− 2.924661366	Down
*Creld2*	1.83504E−43	− 3.736802324	Down
*Pdia6*	1.69019E−42	− 2.851637855	Down
*Manf*	2.08717E−41	− 2.86686964	Down
*Herpud1*	1.76854E−39	− 2.679299641	Down
*Pdia3*	4.9069E−37	− 2.245702844	Down

### Joint analysis of mRNA m^6^A methylation and expression differences after H/R treatment

**Fig. 7:**
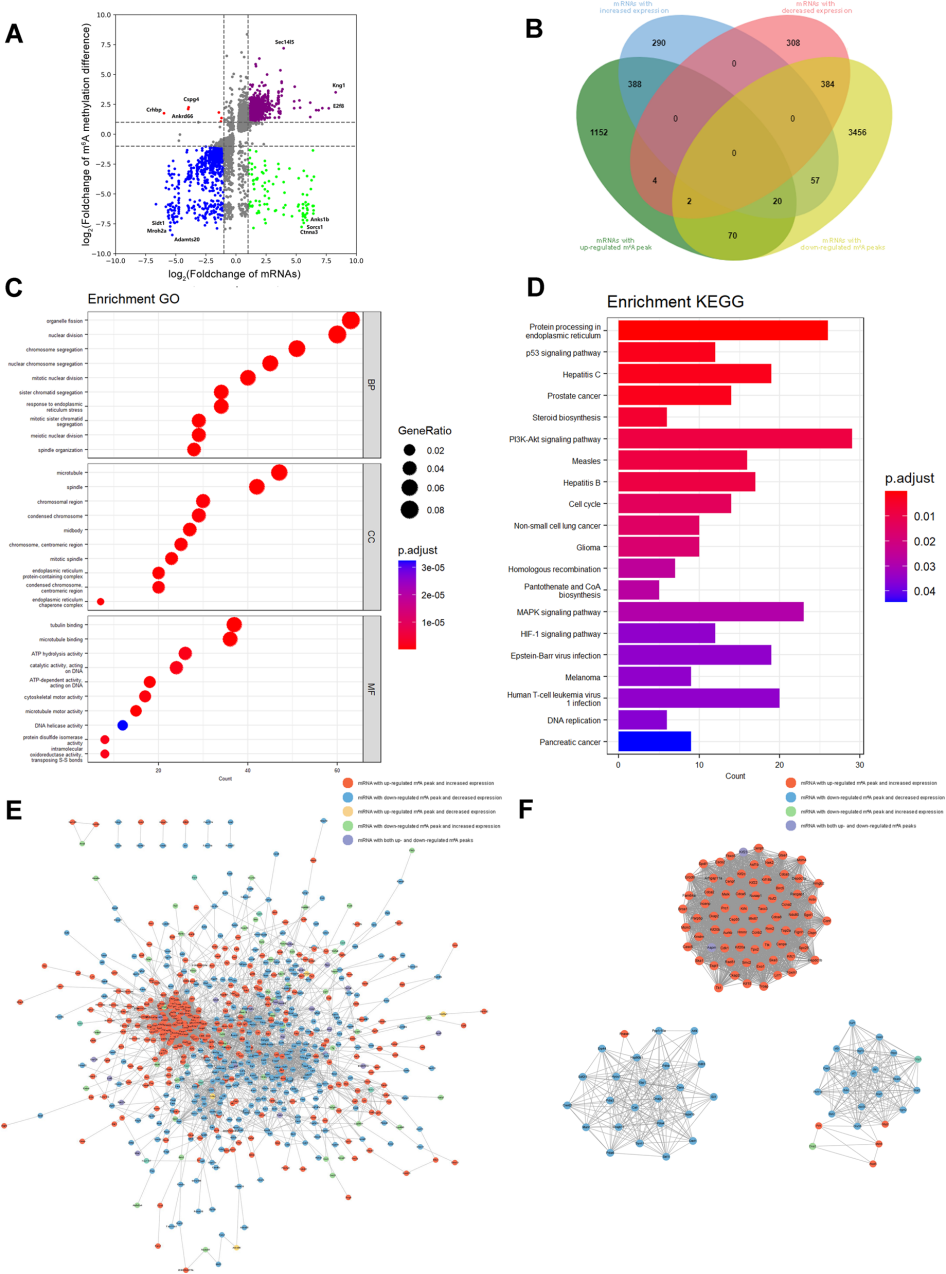
Joint analysis of mRNAs with m^6^A methylation difference and expression difference. **A** Four quadrant plot showing mRNAs with both m^6^A methylation difference and expression difference. **B** Venn plot showing the number of mRNAs with both m^6^A methylation difference and expression difference. **C**, **D** GO and KEGG analysis of the source genes of mRNAs with both m^6^A methylation difference and expression difference. **E** PPI network showing the interactions between mRNAs encoding proteins with both m^6^A methylation differences and expression differences. **F** Three clusters with the highest score in PPI network

Differentially methylated and differentially expressed mRNAs were analyzed. A total of 1068 methylation peaks and their mRNA expression were upregulated, and 767 were downregulated. Six methylation peaks were upregulated, and their mRNA expression levels were downregulated, whereas 98 methylation peaks were downregulated, and their mRNA expression levels were upregulated. Therefore, after joint analysis and duplication according to the gene symbols, there were 408 mRNAs with both m^6^A methylation and expression upregulated, 387 with both downregulated, 6 with m^6^A methylation upregulated and expression downregulated, and 77 with m^6^A methylation downregulated and expression upregulated (Additional file 11: Table S10, Fig. 7A, B). We performed GO and KEGG analyses on mRNAs with both m^6^A methylation and expression changes (Additional files 12, 13: Tables S11, S12, Fig. 7C, D). Consistent with previous enrichment analyses, most of these genes were enriched in terms related to cell division. In addition, we mapped the PPI network to show the relationship between genes that exhibited changes in both m^6^A methylation and expression levels (Fig. 7E). According to MCODE analysis, we screened 25 hub gene clusters (Additional file 14: Table S13) and identified three gene clusters with the highest scores (Fig. 7F). In addition, based on the 12 algorithms of cytoHubba, we identified that cyclin-dependent kinase 1 (*Cdk1*), marker of promotion Ki-67 (*Mki67*), DNA topoisomerase II alpha (*Top2a*), aurora kinase B (*Aurkb*), cyclin B2 (*Ccnb2*), ribbon reduce regulatory bundle M2 (*Rrm2*), and cyclin A2 (*Ccna2*) were the most common hub genes (Table 4).

**Table 4:** The top 20 hub genes identified by 12 algorithms based on cytoHubba analysis

MCC	DMNC	MNC	Degree	EPC	BottleNeck	EcCentricity	Closeness	Radiality	Betweenness	Stress	ClusteringCoefficient
*Rbl1*	*Spdl1*	*Cdk1*	*Cdk1*	*Cdca8*	*Cdk1*	*Cdk1*	*Brca1*	*Brca1*	*Brca1*	*Brca1*	*Mtfr2*
*Psrc1*	*Fam64a*	*Aurkb*	*Brca1*	*Kif20a*	*Mki67*	*Mki67*	*Mki67*	*Mki67*	*Mki67*	*Mki67*	*Zyg11b*
*E2f7*	*Spc25*	*Brca1*	*Aurkb*	*Kif4*	*Stat1*	*Stat1*	*Cdk1*	*Cdk1*	*Stat1*	*Stat1*	*Trim34a*
*Mtfr2*	*Knstrn*	*Mki67*	*Mki67*	*Rrm2*	*Fos*	*Fos*	*Aurkb*	*Hspa1b*	*Fos*	*Cdk1*	*Kif24*
*E2f2*	*Mastl*	*Ccna2*	*Ccna2*	*Brca1*	*Vegfa*	*Vegfa*	*Ccna2*	*Ccna2*	*Cdk1*	*Hspa1b*	*Fgfbp1*
*Brip1*	*Gsg2*	*Top2a*	*Top2a*	*Tpx2*	*Hspa1b*	*Hspa1b*	*Top2a*	*Aurkb*	*Hspa1b*	*Fos*	*Oscar*
*Polq*	*Ska1*	*Cdca8*	*Ccnb2*	*Cdca5*	*Ddx58*	*Atf4*	*Ccnb2*	*Top2a*	*Hsp90b1*	*Hsp90b1*	*Angpt4*
*Cdk6*	*Ckap2l*	*Ccnb2*	*Cdca8*	*Kif23*	*Mapt*	*Hyou1*	*Exo1*	*Stat1*	*Mapt*	*Vegfa*	*Sp110*
*Tk1*	*Ckap2*	*Mcm3*	*Mcm3*	*Kif2c*	*Atf4*	*Vdr*	*Rrm2*	*Ccnb2*	*Vegfa*	*Aurkb*	*Tubb4b-ps1*
*Pola2*	*Ska3*	*Exo1*	*Rrm2*	*Ccna2*	*Hyou1*	*Eprs*	*Mcm3*	*Hsp90b1*	*Pik3r3*	*Nfkbia*	*Pnp*
*Mybl2*	*Nusap1*	*Cdca5*	*Exo1*	*Aurkb*	*Vdr*	*Rrm2*	*Rad51*	*Fos*	*Eprs*	*Top2a*	*Slc8a2*
*Rpa2*	*Depdc1a*	*Rad51*	*Cdca5*	*Cdca2*	*Eprs*	*Top2a*	*Cdca8*	*Exo1*	*Rnasel*	*E2f7*	*Klra4*
*Mcm10*	*Troap*	*Kif2c*	*Rad51*	*Rad51*	*Rrm2*	*Rnasel*	*Birc5*	*Rrm2*	*Top2a*	*Pik3r3*	*Arrdc3*
*E2f8*	*Fbxo5*	*Ttk*	*Kif2c*	*Ttk*	*Top2a*	*Ccnb2*	*Melk*	*Nfkbia*	*Nfkbia*	*Mapt*	*Selplg*
*Eme1*	*Tacc3*	*Ndc80*	*Ttk*	*Cep55*	*Rnasel*	*Cdkn1a*	*Ttk*	*Igf1*	*Ldlr*	*Rnasel*	*Nlrp6*
*Aunip*	*Depdc1b*	*Rrm2*	*Ndc80*	*Asf1b*	*Ldlr*	*Smc2*	*Foxm1*	*Vegfa*	*Kitl*	*Exo1*	*Patz1*
*Lmnb1*	*Fignl1*	*Melk*	*Melk*	*Prc1*	*Sspo*	*Hmox1*	*Cdca5*	*Mcm3*	*Aurkb*	*Ccna2*	*Ddx59*
*Chaf1a*	*Anln*	*Birc5*	*Birc5*	*Cenpf*	*Ccnb2*	*Racgap1*	*Tpx2*	*Rad51*	*Igf1*	*Igf1*	*Zbtb14*
*Tcf19*	*Fam83d*	*Kif20a*	*Kif20a*	*Mcm3*	*Lrr1*	*Xbp1*	*Kif2c*	*Birc5*	*Atf4*	*Canx*	*Bahcc1*
*Dsn1*	*Kifc1*	*Kif4*	*Kif4*	*Cdk1*	*Cdkn1a*	*Pik3r3*	*Kif4*	*Foxm1*	*Aldh18a1*	*Ccnb2*	*Cd93*

### Knockdown of *Mettl14* inhibited the proliferation of NSCs

**Fig. 8:**
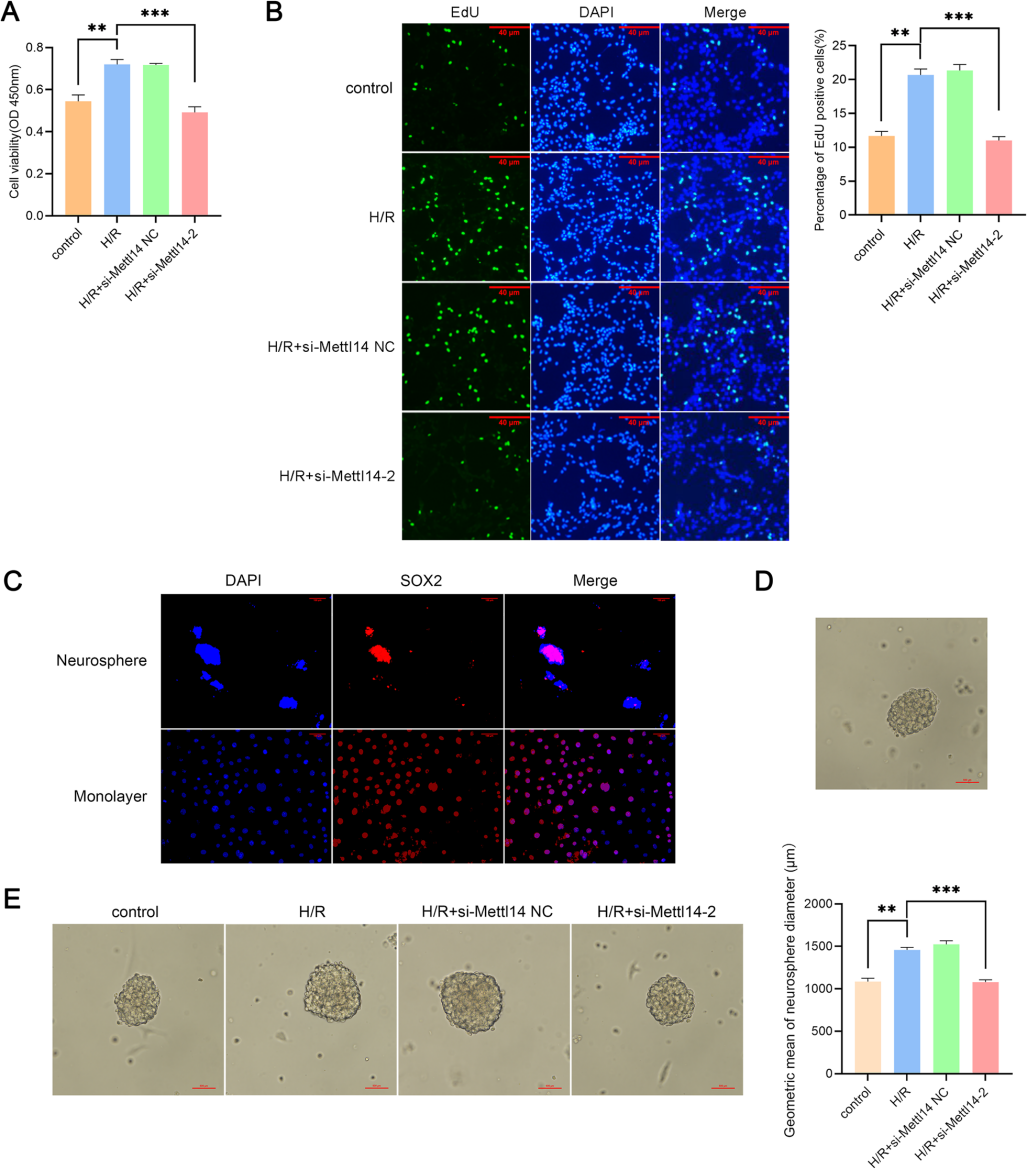
Knockdown of *Mettl14* inhibited the proliferation of NSCs after H/R. **A** Detection of the cell viability of NSCs that undergo H/R treatment after *Mettl14* knockdown by CCK-8 assay (n = 3). **P < 0.01; ***P < 0.001. **B** The EdU-positive cells in NSCs treated with H/R after *Mettl14* knockdown were observed by immunofluorescence staining (n = 3). Scale bar = 40 μm. **P < 0.01; ***P < 0.001. **C** NSCs in suspended and adherent states. **D** The cell spheroidizing ability of NSCs. **E** Detection of the geometric mean diameter of neurosphere that undergo H/R treatment after *Mettl14* knockdown by cell spheroidization assay (n = 3). **P < 0.01; ***P < 0.001. The data are presented as the mean ± SEM

Based on enrichment analysis of the RNA-seq results, we verified the proliferation of NSCs after H/R. The results of the joint analysis also showed that changes in m^6^A methylation of some mRNAs were related to proliferation. After H/R treatment, the viability of NSCs increased (*P* = 0.0038, Foldchange = 1.3225) (Fig. 8A), and the number of EdU-positive cells increased (*P* = 0.0012, Foldchange = 1.7712) (Fig. 8B). However, when *Mettl14* was knockdown, these trends were reversed (*P* = 0.0007 and *P* = 0.0008, respectively) (Fig. 8A, B). In addition, mouse primary NSCs were extracted and identified for cell spheroidization experiments (Fig. 8C, D). Compared with the control group, the geometric average diameter of neurospheres in the H/R group significantly increased (*P* = 0.0015, Foldchange = 1.3429), and this trend was eliminated after knocking down *Mettl14* (*P* = 0.0007) (Fig. 8E).

## Discussion

Ischemia–reperfusion injury of the nervous system often causes serious complications, such as hemiplegia after ischemic stroke and paraplegia after spinal cord blood supply is blocked during aortic surgery [3, 27]. A number of recent studies have reported on therapeutic strategies and molecular mechanisms to promote nerve repair after ischemia–reperfusion injury, among which activating endogenous NSCs seems most promising in terms of avoiding immune rejection and other aspects [11, 12]. In addition, because of the universality and reversibility of mRNA m^6^A methylation in eukaryotes, its role in various pathophysiological processes has attracted attention. In this study, NSCs were treated with H/R in vitro to simulate the activation of endogenous NSCs following ischemia–reperfusion injury in vivo. We found that after experiencing H/R, the m^6^A methylation modification level of mRNAs in NSCs significantly changed, and *Mettl14* mRNA expression levels were affected by H/R, indicating that *Mettl14* plays a key role in m^6^A methylation of mRNA in NSCs after H/R. Next, we innovatively implemented MeRIP-Seq and RNA-seq simultaneously, explored the effects of m^6^A methylation modification and mRNA expression changes on the biological activities of NSCs after H/R, and verified the endogenous proliferation and migration of NSCs after ischemia–reperfusion injury using bioinformatics analysis. In addition, we identified mRNAs with altered expression due to the change in m^6^A methylation after H/R and identified hub genes.

As a widely studied methyltransferase*, Mettl14* could affect cell proliferation by regulating m^6^A methylation modification of forkhead box O 3a (*Foxo3a*) [28]. In a study of carotid atherosclerosis, a common cause of ischemic stroke, *Mettl14* increased the m^6^A methylation of *Foxo1* mRNA, which promoted *Foxo1* translation and transcription factor activity to increase endothelial inflammation [29]. In embryonic NSCs, knockout of *Mettl14* led to changes in histone modification across the whole genome, thus affecting gene expression and reducing the proliferation of NSCs [19]. *Mettl14* was also shown to be an essential factor for axonal regeneration of dorsal root ganglion neurons [30]. It has been suggested that the m^6^A methylation level of mRNAs in NSCs might increase after H/R. In this study, of the five methyltransferases or demethylases tested, *Mettl14* was the only one with different expression levels in NSCs after H/R. The sequencing results showed that the number of mRNA peaks in the H/R group was significantly higher than that in the control group, which also supports this view (Fig. 2D). The results of previous studies and the present study indicate that m^6^A methylation can regulate the proliferation of NSCs [18, 19]. Therefore, we speculated that *Mettl14* plays an important role in the H/R-mediated activation of NSCs. Subsequent experiments also confirmed that *Mettl14* was involved in the migration and proliferation of NSCs after H/R. Future research should continue to focus on mRNA targets that undergo *Mettl14*-promoted m^6^A methylation to further reveal the molecular mechanism of endogenous NSC activation.

It has been reported that after cerebral ischemia–reperfusion injury, NSCs are endogenously activated and proliferate [14–17]. Undoubtedly, cell proliferation is a prerequisite for subsequent nerve repair. In this study, we discovered mRNAs whose expression levels change with the methylation of m^6^A after the joint analysis of MeRIP-seq and RNA-seq results. Enrichment analysis of these genes showed that they were significantly related to cell division. We then analyzed and identified the seven most common hub genes. *Cdk1*, *Ccnb2*, and *Ccna2* are considered the core components of the cell cycle regulation system. A previous study showed that *Cdk1* could be regulated by histone deacetylase 3, controlling G2/M phase progression and mitosis of NSCs [31], and knockout of *Ccna2* could cause abnormal DNA repair [32]. *Aurkb* is a serine/threonine kinase that is essential for G2/M phase transition [33]. *Mki67* encodes a nuclear protein related to cell proliferation that can be used as a marker of cell proliferation, and *Top2a* encodes an enzyme protein closely related to cell proliferation, apoptosis, and mitosis [34, 35]. A study showed that *Top2a* can be used as a characteristic gene to distinguish NSCs from astrocytes [36]. However, there are few studies on the regulation of NSC proliferation by these seven hub genes, and further exploration is needed in the future.

Neural repair is a complex process, and the observation of endogenous proliferation of NSCs alone does not represent the neural repair process. The migration of NSCs to the injured site and differentiation to replace injured neurons are the next steps to complete nerve repair [37, 38]. Although the results of our joint MeRIP-seq and RNA-seq analysis were not related to migration or differentiation, we could find that the source genes of mRNAs with m^6^A methylation differences were related to cell adhesion and synapse formation through separate enrichment analysis of the MeRIP-seq results. In the process of migration to other tissues, cells must constantly interact with other cells and produce dynamic changes in adhesion and detachment. The formation of synaptic connections is regarded as the last step in nerve repair and the functional embodiment of NSCs after differentiation into neurons. We also verified that H/R could significantly improve the migration ability of NSCs. Due to the differences in the results of these enrichment analyses, we speculated that m^6^A methylation regulates the migration and differentiation of NSCs through other mechanisms, such as enhancing RNA translation efficiency rather than affecting mRNA expression. Further research into the hub genes from genes enriched in relevant terms should help elucidate the molecular mechanism that regulate NSC migration and differentiation through m^6^A methylation modification after H/R.

## Conclusion

In conclusion, this study is the first to describe the m^6^A methylation and expression profiles of mRNAs in NSCs after H/R. Bioinformatics analysis revealed that proliferation, migration, and differentiation of NSCs after H/R are closely related to m^6^A methylation of mRNAs. *Mettl14* plays an important role in cell proliferation and migration. This provides a reference for studying the specific biological processes and molecular mechanisms of endogenous activation of NSCs after ischemia–reperfusion injury in the central nervous system.

### Supplementary Information

**Additional file 1: Table S1.** The detailed information of raw data.

**Additional file 2: Table S2.** Statistical analysis of reads mapped in reference genome.

**Additional file 3: Table S3.** The detailed information of m^6^A peaks.

**Additional file 4: Table S4.** The detailed information of significantly changed m^6^A peaks.

**Additional file 5: Table S5.** Gene ontology analysis of the source genes of mRNAs with m^6^A methylation differences.

**Additional file 6:** **Figure S1.** Protein–protein interaction network for enrichment analysis results of the source genes of mRNAs with m^6^A methylation differences. **A** Genes enriched in the GO analysis term “synapse organization.” **B** Genes enriched in the GO analysis term “extracellular structure organization.” **C** Genes enriched in the GO analysis term “cell adhesion molecule binding.” **D** Genes enriched in the KEGG analysis term “ECM-receptor interaction.” E Genes enriched in the KEGG analysis term “axon guidance.”

**Additional file 7: Table S6.** Kyoto encyclopedia of genes and genomes analysis of the source genes of mRNAs with m^6^A methylation differences.

**Additional file 8: Table S7.** The detailed information of significantly changed mRNAs.

**Additional file 9: Table S8.** Gene ontology analysis of differentially expressed genes.

**Additional file 10: Table S9.** Kyoto encyclopedia of differentially expressed genes.

**Additional file 11: Table S10.** The detailed information of conjoint analysis between m^6^A methylation and RNA expression

**Additional file 12: Table S11.** Gene ontology analysis of the source genes of mRNAs with m^6^A methylation difference and expression difference.

**Additional file 13: Table S12.** Kyoto encyclopedia of genes and genomes analysis of the source genes of mRNAs with m^6^A methylation differences.

**Additional file 14: Table S13.** Hub gene clusters based on MCODE analysis.

**Additional file 15: Figure S2.** Full-length blots of Fig. 5B.

**Additional file 16: Figure S3.** The fluorescence staining of transfection efficiency in NSCs.

## Data Availability

The datasets generated and/or analyzed during the current study are available in the GEO (Gene Expression Omnibus) repository under the accession number GSE221841 (https://www.ncbi.nlm.nih.gov/geo/query/acc.cgi?acc=GSE221841) and GSE221842 (https://www.ncbi.nlm.nih.gov/geo/query/acc.cgi?acc=GSE221842).

## Abbreviations

CNS: Central nervous system
NSCs: Neural stem cells
m^6^A: N6-methyladenosine
H/R: Hypoxia/reoxygenation
qRT-PCR: Quantitative real-time PCR
MeRIP-seq: Methylated RNA immunoprecipitation sequencing
RNA-seq: RNA sequencing
CCK-8: Counting kit-8
FTO: Fat mass and obesity-associated gene
Mettl14: Methyltransferase-like 14
GAPDH: Glyceraldehyde phosphate dehydrogenase
GO: Gene ontology
KEGG: Kyoto Encyclopedia of Genes and Genomes
PPI: Protein–protein interaction
NC: Negative control
OD: Optical density
SEM: Standard error of the mean
METTL3: Methyltransferase-like 3
WTAP: WT1 associating protein
ALKBH5: AlkB homolog 5
Cdk1: Cyclin-dependent kinase 1
Mki67: Marker of promotion Ki-67
Top2a: DNA topoisomerase II alpha
Aurkb: Aurora kinase B
Ccnb2: Cyclin B2
Rrm2: Ribbon reduce regulatory bundle M2
Ccna2: Cyclin A2
Foxo3a: Forkhead box O 3a
Foxo1: Forkhead box O 1
