# Supplementary figures and images for: The m6A methylation and expression profiles of mouse neural stem cells after hypoxia/reoxygenation
Source: Stem Cell Res Ther. 2024 Feb 16;15:43. doi: 10.1186/s13287-024-03658-8 (PMC10870567; doi:10.1186/s13287-024-03658-8)

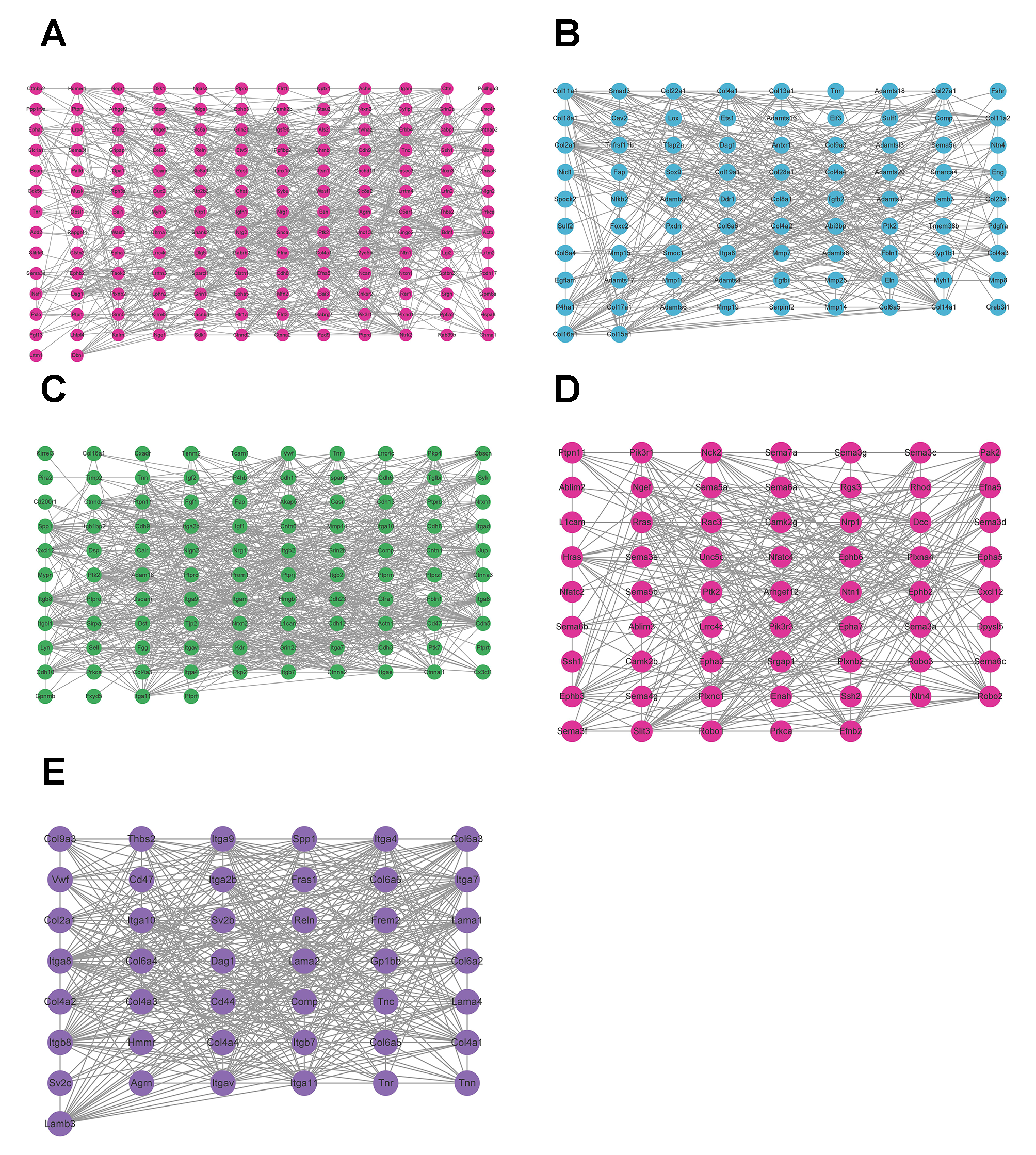

Supplement: Supplementary file 6 — Additional file 6: Figure S1. Protein–protein interaction network for enrichment analysis results of the source genes of mRNAs with m6A methylation differences. A Genes enriched in the GO analysis term “synapse organization.” B Genes enriched in the GO analysis term “extracellular structure organization.” C Genes enriched in the GO analysis term “cell adhesion molecule binding.” D Genes enriched in the KEGG analysis term “ECM-receptor interaction.” E Genes enriched in the KEGG analysis term “axon guidance.” [file 13287_2024_3658_MOESM6_ESM.tif]

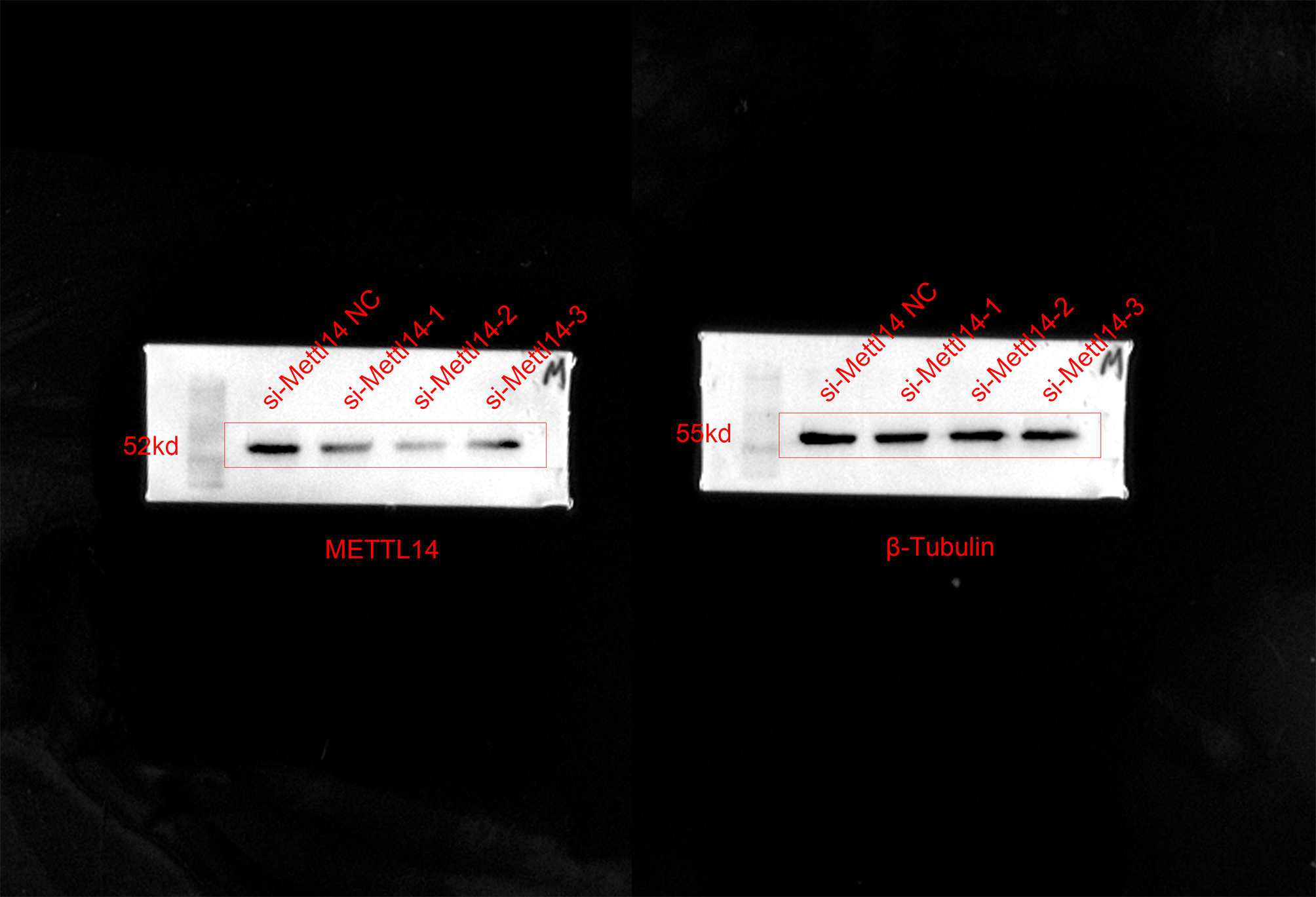

Supplement: Supplementary file 15 — Additional file 15: Figure S2. Full-length blots of Fig. 5B. [file 13287_2024_3658_MOESM15_ESM.tif]

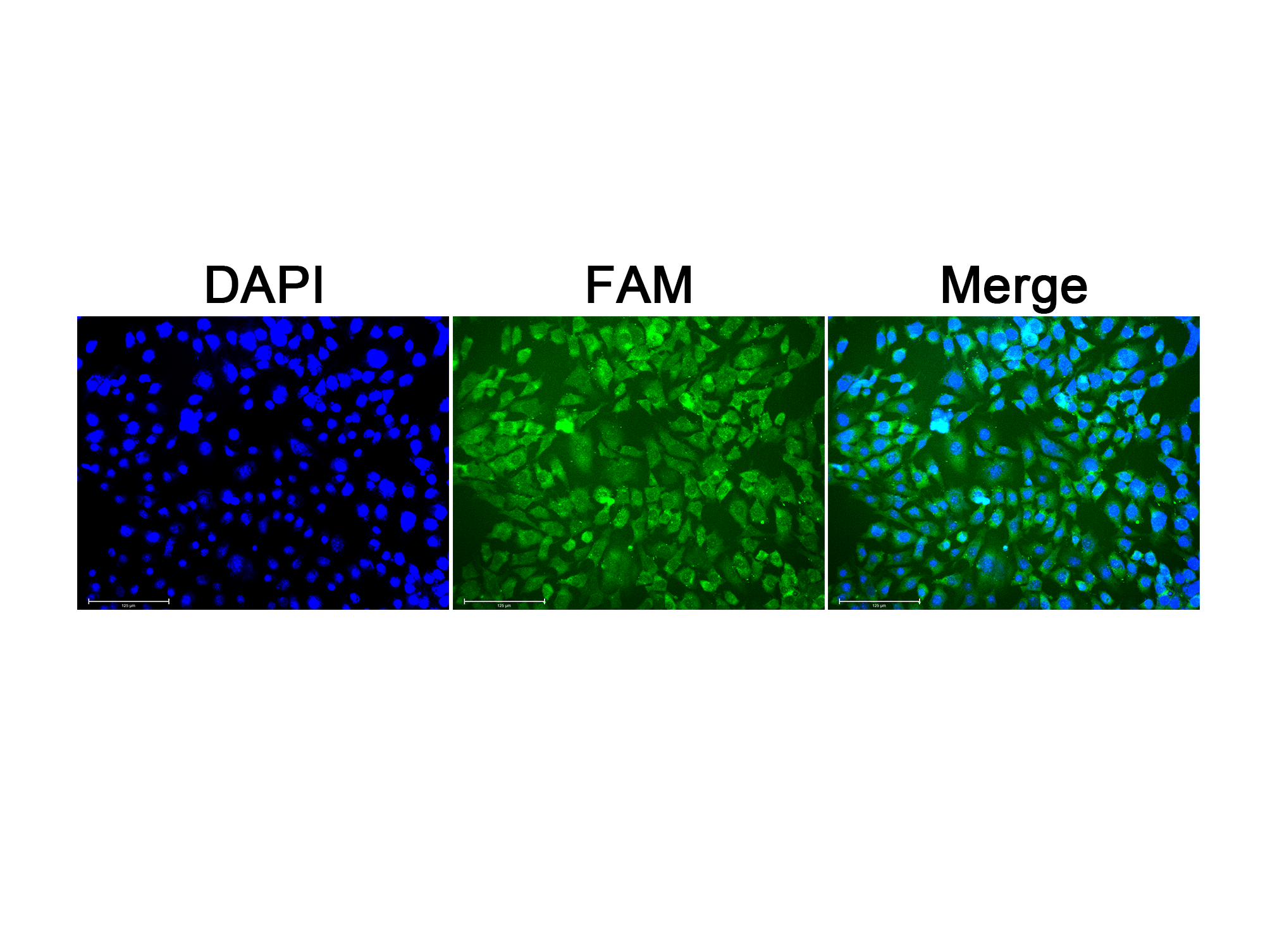

Supplement: Supplementary file 16 — Additional file 16: Figure S3. The fluorescence staining of transfection efficiency in NSCs. [file 13287_2024_3658_MOESM16_ESM.tif]
